# Hidden and overall haemorrhage following minimally invasive and open transforaminal lumbar interbody fusion

**DOI:** 10.1007/s10195-017-0464-9

**Published:** 2017-07-24

**Authors:** Yang Yang, Liangming Zhang, Bin Liu, Mao Pang, Peigen Xie, Zihao Chen, Wenbin Wu, Feng Feng, Limin Rong

**Affiliations:** Department of Spine Surgery, The Third Affiliated Hospital of Sun Yat-sen University, 600 Tianhe Road, Tianhe District, Guangzhou, Guangdong Province China

**Keywords:** Haemorrhage, Hidden, Transforaminal approach, Lumbar interbody fusion, Minimally invasive surgery

## Abstract

**Background:**

Hidden haemorrhage has been proved to be significant in joint surgery. However, when referring to lumbar interbody fusion, it is often ignored because of its invisibility. This randomized controlled study aimed to calculate and compare hidden haemorrhage following minimally invasive and open transforaminal lumbar interbody fusion (MIS-TLIF and open TLIF). Meanwhile, its clinical significance was also analyzed.

**Materials and methods:**

A total of 41 patients were included in this study, then they were randomized to receive MIS-TLIF or open TLIF, 21 and 20, respectively. For each case, total volume loss of red blood cell (RBC) was calculated by Gross' formula based on perioperative haematocrit change, then perioperative visible volume loss of RBC was calculated through haemorrhage volume and weight. After deducting it from total volume loss of RBC, hidden volume loss of RBC was obtained. Absolute amount of hidden haemorrhage and its ratio upon total haemorrhage, as well as indicators assessing clinical outcomes, including visual analogue scale (VAS) for back and leg, Oswestry disability index (ODI), interbody fusion rate and complication incidence were compared and analyzed.

**Results:**

Mean hidden volume loss of RBC in MIS-TLIF was significantly reduced compared with open TLIF (166.7 versus 245.6 ml). Besides, both mean total and visible volume loss of RBC in MIS-TLIF were also statistically less than those in open TLIF (355.3 versus 538.6 ml; 188.6 versus 293.0 ml). While mean ratio of hidden haemorrhage upon total haemorrhage was 46.7% for MIS-TLIF and 44.5% for open TLIF, respectively, showing no statistical significance. At one week postoperatively, more significant improvements of VAS for back and leg, as well as ODI were seen in MIS-TLIF compared with open TLIF. While at final follow-up of at least 2 years, all parameters continued to improve and revealed no statistical difference between both surgeries. Similar interbody fusion rate and complication incidence were observed in both series.

**Conclusions:**

Besides reduced visible haemorrhage and improved clinical outcomes, MIS-TLIF also owns the superiority of less hidden haemorrhage, offering another advantage over open TLIF.

**Level of evidence:**

Level II.

## Introduction

Minimally invasive transforaminal lumbar interbody fusion (MIS-TLIF) has been demonstrated as one favorable alternative for treatment of spinal degenerative diseases owing to the advantages of reduced surgical trauma, minimized intraoperative haemorrhage, enhanced postoperative recovery and improved cost-utility [1–4], whereas the actual amount of perioperative haemorrhage is considerably larger than the measured volume, which had been first proved and detailed by Sehat et al. in 2000 [5]. Since then, more considerations have been given to hidden haemorrhage and its contributing factors in an attempt to avoid associated complications, involving massive allogeneic blood transfusion and postoperative severe anemia. Up to now, some studies investigating hidden haemorrhage after total knee arthroplasty (TKA) or total hip arthroplasty (THA) were reported [6–8]. To the best of our knowledge, there are few studies focusing on calculation and comparison of hidden haemorrhage between MIS-TLIF and open TLIF, as well as analyzing its clinical importance. In this series, one randomized controlled study was conducted to evaluate absolute amount of hidden haemorrhage and its ratio upon total haemorrhage following MIS-TLIF and open TLIF.

## Materials and methods

### Clinical data

A total of 41 patients revealing bilateral neurological symptoms with the single operated segment of L4/5 were included in this research. Inclusion criteria were as follows: segmental instability at the level of spinal stenosis, huge lumbar disc herniation with segmental instability, either grade 1 or 2 spondylolisthesis based on Meyerding classification [9, 10]. While exclusion criteria were as follows: previous spinal instrumentation, spinal tumor pathology or infection, acute spinal fracture, revealing chronic liver disease, hematologic disease or laboratory sign of bleeding disorder, showing very large intraoperative blood loss, and the trigger was 1500 ml [8, 10]. All included cases were refractory to standard conservative treatments, such as medications and physical therapies for at least six weeks. In this study, twenty-one patients were randomly assigned to receive MIS-TLIF (minimally invasive group, MIS group) and the remaining 20 patients underwent open TLIF randomly (open group). One senior surgeon performed all minimally invasive and open surgeries, meanwhile, all these cases were on the plateau stage of the surgeon’s learning curve for either surgical technique. This study was approved by institutional ethic committee of the hospital and informed consents were obtained from all participants.

### Surgical procedures for MIS-TLIF

Following general anaesthesia, patient was evenly positioned prone on radiolucent table. G-arm X-ray machine was used to confirm operated level. After sterilizing and draping of skin, four paracentral mini-incisions were made about 4.5 cm lateral to the midline. Under fluoroscopy guidance, Jamshidi needle was accurately inserted into vertebrae through pedicle route, then its inner stylet was removed to allow placement of kirschner wire. Fixed-diameter working channel with 20-mm diameter (METRx II) was inserted down to the zygapophyseal joint following sequentially placing dilators over each other through the incision. Partial facetectomy was performed using rongeur from lateral to medial to expose posterolateral part of spinal canal. The rest of ipsilateral facet, lamina and lateral margin of ligamentum flavum were removed to accomplish sufficient canal decompression, then working channel was tilted to contralateral side where indicated through the same incision to perform further canal decompression. After thorough discectomy and well preparation of endplates, autologous bone graft from resected local bony structures, including facet and lamina were packed in disc space, and interbody cage (polyetheretherketone, PEEK) filled with it was also placed medially, then screws and rods were inserted percutaneously. Bilateral compression was applied before final tightening of the pedicle screw-rod construct. Finally, closure in layers was performed following wound haemostasis and irrigation.

### Surgical procedures for open TLIF

Through a midline incision, the fascia was incised and paraverterbral muscles were mechanically detached from the bony structure. After confirmation of targeted level, bilateral pedicle screws were inserted, followed by bilateral laminotomy and facetectomy to accomplish adequate canal decompression. Then, discectomy was performed and endplates were well prepared through curetting. After medial placement of autologous bone graft and proper interbody cage filled with it, rods were inserted and finally tightened. Haemostasis and irrigation were done before closing the wound.

### Postoperative managements

The drainage tube was removed at 24 h postoperatively and the drainage volume was recorded. During the first 48 h after operation, adequate administration of analgesics and intravenous fluids should be guaranteed to maintain circulation stability. However, low molecular weight heparin was not routinely used for the concern of intraspinal haematoma. Instead, antithrombotic compression stocking and intermittent foot pump were initiated within several hours following surgery. When motion function of bilateral lower extremities improved, active ambulation was encouraged to prevent deep vein thrombosis.

### Calculation of hidden haemorrhage

As perioperative haemorrhage occurs, circulation volume of patient tends to fall. However, adequate and simultaneous administration of venous fluids can supplement the blood loss, maintaining stable circulation. In 1983, Gross developed the linear formula using average haematocrit during perioperative course to calculate blood loss [11]. When using Gross' formula, cases undergoing tremendous or brisk haemorrhage should be excluded, for the actual volume of haemorrhage is largely inconsistent with that calculated by Gross' formula [12]. Meanwhile, all included cases receiving simultaneous and balanced venous infusion perioperation are considered hemodynamically stable through well-maintained signs, such as heart rate, peripheral blood pressure and central venous pressure, and hematocrit of wound drainage is expected nearly the same as that of peripheral venous blood.

According to methodology introduced by Sehat et al. [12], we modified some procedures in this study. All patients received a series of full blood routine tests to obtain haematocrit preoperation (HCT_pre_), on operative day and postoperation (consecutive five days). The height and body weight of all patients were recorded and thus, patient’s blood volume (PBV) could be calculated by the formula introduced by Nadler et al. [13]:

$${\text{PBV }} = \, k_{ 1} \times {\text{height }}\left( {\text{m}} \right)^{3} + \, k_{2} \times {\text{weight }}\left( {\text{kg}} \right) \, + \, k_{3} ,$$

where *k*_l_ = 0.3669, *k*_2_ = 0.03219, *k*_3_ = 0.6041 for men; *k*_l_ = 0.3561, *k*_2_ = 0.03308, *k*_3_ = 0.1833 for women.

**Fig. 1:**
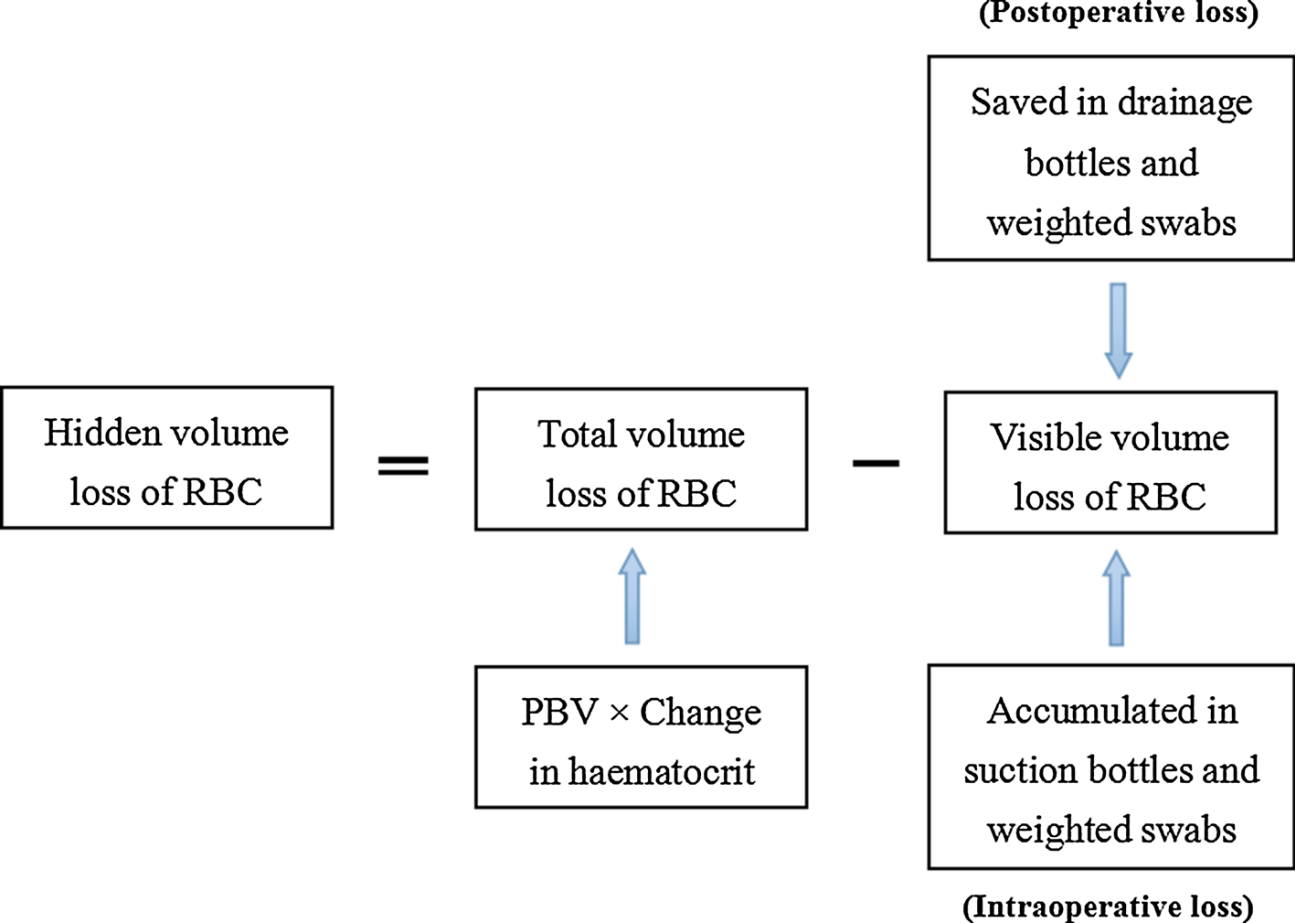
Flowchart for calculating hidden haemorrhage

During consecutive 5 days postoperation, the lowest HCT value was recorded as HCT_post_. For perioperative haemodynamical stability was well maintained, multiplying PBV by the deduction between HCT_pre_ and HCT_post_ (HCT_pre_ − HCT_post_) gave the total volume loss of red blood cell (RBC). Visible volume loss of RBC is composed of volume loss intraoperation and postoperation. The weight of lost blood accumulated in suction bottles and swabs during operation was evaluated and then it was converted to the volume by dividing density of blood. The arithmetic product of haemorrhage volume and HCT_ave_ (the arithmetic mean value of HCT_pre_ and haematocrit on operative day) was considered as volume loss of RBC during operation. Similarly, postoperative volume loss of RBC was the sum of those accumulated in drainage bottle and weighted swabs. As haematocrit of drainage was considered as the same as that of peripheral venous blood, multiplying volume of drainage by corresponding peripheral haematocrit gave the volume loss of RBC from drainage. After adding volume loss of RBC saved in weighted swabs, postoperative volume loss of RBC was able to be calculated. Hence, hidden volume loss of RBC was deemed as deduction between total and visible volume loss of RBC (Fig. 1).

### Statistical analysis

Statistical analysis was performed using SPSS version 17.0. Independent *t* test was used to compare continuous variables (expressed as mean ± standard deviation), including baseline data, visual analogue scale (VAS) for back and leg, Oswestry disability index (ODI), surgical duration, as well as total, visible, hidden volume loss of RBC and the ratio of hidden haemorrhage. Chi squared test was applied to compare categorical data, involving gender, preoperative diagnose, interbody fusion rate and complication incidence. In this study, statistical significance was defined as *P* < 0.05.

## Results

For preoperative baseline data, no statistical difference was observed between both groups (all *P* > 0.05, Table 1). All patients underwent single-level operation smoothly without blood transfusion. In Table 2, hidden volume loss of RBC in MIS group was significantly less than that in open group (166.7 versus 245.6 ml, *P* = 0.028). Similarly, there were obviously less total and visible volume loss of RBC in MIS group versus open group (MIS 355.3 and 188.6 ml, open 538.6 and 293.0 ml, both *P* < 0.001). However, with respect to the ratio of hidden haemorrhage upon total haemorrhage, there was no significant difference between both groups (MIS 46.7%, open 44.5%, *P* = 0.626).

**Table 1:** Comparison of preoperative baseline data between minimally invasive and open group

	Minimally invasive TLIF	Open TLIF	*P* value
Age (year-old)	63.5 ± 9.1	58.0 ± 10.9	0.195
Gender (M/F)	7/14	8/12	0.658
BMI (kg/m^2^)	23.7 ± 2.9	25.0 ± 3.2	0.322
Disease etiology			
Spinal stenosis	11	9	0.636
Spondylolisthesis	5	6	0.655
Disc herniation with segmental instabili	5	5	1.000
VAS (back)	5.8 ± 0.9	5.6 ± 0.8	0.562
VAS (leg)	5.2 ± 1.3	4.9 ± 1.8	0.708
ODI (%)	43.5 ± 15.1	44.2 ± 14.3	0.916
Preoperative haematocrit	0.403 ± 0.038	0.426 ± 0.049	0.203
PBV (L)	3.75 ± 0.75	4.20 ± 0.67	0.132

*TLIF* transforaminal lumbar interbody fusion, *BMI* body mass index, *VAS* visual analogue scale, *ODI* Oswestry disability index, *PBV* patient’s blood volume

**Table 2:** Comparison of perioperative volume loss of RBC between minimally invasive and open group

	Minimally invasive TLIF	Open TLIF	*P* value
Total volume loss of RBC (ml)	355.3 ± 75.0	538.6 ± 129.5	<0.001
Visible volume loss of RBC (ml)	188.6 ± 42.3	293.0 ± 78.9	<0.001
Hidden volume loss of RBC (ml)	166.7 ± 48.8	245.6 ± 97.0	0.028
Ratio of hidden haemorrhage (%)	46.7 ± 7.8	44.5 ± 12.7	0.626

*RBC* red blood cell

When referring to clinical outcomes, surgical duration of MIS-TLIF was longer than that of open TLIF (179.0 versus 141.8 min, *P* < 0.001). At 1 week postoperatively, VAS of both back and leg in MIS group were more reduced than those in open group (back: 2.4 versus 3.4, *P* < 0.001, leg: 2.8 versus 3.5, *P* = 0.004), and MIS group also had more decreased ODI than open group (27.3 versus 35.2%, *P* = 0.003). All patients were regularly followed up for at least 2 years. At final follow-up, VAS of back and leg, as well as ODI for both groups continued to improve compared with those one week postoperation. While comparing each of these parameters between MIS and open group, all revealed no statistical significance (VAS-back: 1.0 versus 1.2, *P* = 0.471, VAS-leg: 0.6 versus 0.9, *P* = 0.371, ODI: 12.0 versus 13.5%, *P* = 0.461). According to Bridwell criteria [14], interbody fusion rate of Grade I between both groups was nearly the same (MIS 85.7%, open 80%, *P* = 0.943). There were two cases of incidental dural tear in MIS group and one case of delayed wound healing in open group, revealing similar complication incidence (MIS 9.5%, open 5%, *P* = 1.000). All three patients got full recovery following conservative treatment or surgical debridement. No instrumentation-related complication, such as neurological injury or implant loosening was found in either group (Table 3).

**Table 3:** Comparison of intraoperative and postoperative data between minimally invasive and open group

	Minimally invasive TLIF	Open TLIF	*P* value
Surgical duration (min)	179.0 ± 20.7	141.8 ± 18.8	<0.001
Interbody fusion (grade I)	18/21	16/20	0.943
Complication	2/21	1/20	1.000
VAS (back)			
One week postoperation	2.4 ± 0.7	3.4 ± 0.9	<0.001
Final follow-up	1.0 ± 0.9	1.2 ± 1.2	0.471
VAS (leg)			
One week postoperation	2.8 ± 0.7	3.5 ± 0.8	0.004
Final follow-up	0.6 ± 0.7	0.9 ± 0.9	0.371
ODI (%)			
One week postoperation	27.3 ± 6.4	35.2 ± 9.4	0.003
Final follow-up	12.0 ± 6.4	13.5 ± 6.5	0.461

## Discussion

Hidden haemorrhage is not usually recognized by general assessment because of its invisibility, while an association is found between increased blood loss and perioperative complications [15]. Hidden haemorrhage can exacerbate postoperative hemoglobin drop, leading to increased transfusion requirement, if not properly managed, it may induce delayed wound healing, increased risk of infection and prolonged postoperative rehabilitation. However, given its significant clinical influence on patients undergoing surgeries, hidden haemorrhage has not received enough attention up to now [6]. With respect to spinal surgeries, elucidating absolute and relative amount of hidden haemorrhage is of great importance in order to avoid aforementioned potential complications. This study first demonstrates that for both MIS-TLIF and open TLIF, there is associated with a certain amount of hidden haemorrhage. At present, since the applications of MIS-TLIF and other kinds of minimally invasive techniques are gaining more popularity because of less iatrogenic injuries [16, 17], it would be speculated that MIS-TLIF is associated with less hidden haemorrhage. This research confirms the hypothesis that absolute amount of hidden haemorrhage in MIS-TLIF is significantly reduced compared with open TLIF, while the ratio of hidden haemorrhage for either surgical technique is nearly the same. For elderly or frail patients requiring canal decompression and lumbar interbody fusion, surgical procedure with less perioperative haemorrhage should be more recommended, and thus MIS-TLIF may be preferred over open surgery. For both MIS-TLIF and open TLIF, especially the latter, simultaneous and sufficient vein infusion is absolutely important after operation because of a relatively larger amount of hidden haemorrhage. It also emphasizes the need for orthopaedic surgeons to be vigilant to the risk of anemia following surgeries. In this study, all included patients were associated with single-level pathology and none of them needed postoperative transfusion because of not so much drop of haemoglobin (The threshold of red blood cell transfusion is less than 70 g/l in our hospital). However, for cases with multiple operated levels or requiring longer surgical duration, those undergoing open surgeries may have more chance of transfusion because of increased visible and hidden haemorrhage.

This study reveals that more surgical time is needed when performing MIS-TLIF, however, less hidden haemorrhage is observed. Thus, it may be speculated that surgical duration is not the main contributor to hidden haemorrhage, which is consistent with the previous study conducted by Shen et al. [6]. However, when performing THA, minimally invasive surgical technique is associated with a larger amount of hidden haemorrhage [8, 18], which is contradictory to our finding. This difference may be explained by less surgical field inside the wound associated with MIS-TLIF procedures, namely reduced and compressed wound surface following suture contributes to decreased hidden haemorrhage. In order to accurately calculate total and hidden haemorrhage, the lowest postoperative haematocrit value is of great importance among several parameters [19]. In this study, haematocrit value of consecutive 5 days following operations were recorded instead of measured haematocrit value 2 or 3 days postoperatively [5, 12]. Based on the data of this research, it could be observed that the majority of included cases presented the lowest haematocrit value during the third to fifth day postoperatively. On the contrary, the amount of patients showing the lowest haematocrit on the second day postoperatively was relatively small. For certain cases, if we use the haematocrit value on the second or third day postoperatively, then calculated amount of total and hidden haemorrhage would be underestimated because of the “falsely” lowest haematocrit value. Previous study has also found that when performing TKA and THA, haemoglobin started to return following 4 days postoperation [6, 19], which is similar to our observations. In our opinion, if the haematorit values of consecutive postoperative 5 days have the tendency to decrease continuously, and the lowest haematocrit value may not appear during this period, prolonging the time frame of blood routine test is needed until the lowest haematocrit value emerges for the purpose of accurate assessment.

However, some drawbacks of this study should be acknowledged. First, it is limited by the small sample size and sole operated level, hence, shortened clinical stringency can be observed in this study. Second, although postoperative haemodynamical stability is well maintained through monitoring of vital signs, there remains the possibility of excessive or insufficient intravenous infusion postoperatively, leading to hypervolemia or hypovolemia. Thus, the calculated total, visible and hidden volume loss of RBC would be overestimated or underestimated. Third, the actual haematorit value of drainage fluid is smaller than that of simultaneous sample from peripheral venous blood [20], so the exact amount of RBC loss in the drainage should be less than indicated by our data, causing statistical bias to some extent. However, all these limitations are not considered to generate obvious deviation to conclusion.

In conclusion, hidden, along with total and visible haemorrhage in MIS-TLIF are significantly less than those in open TLIF, while regarding to the ratio of hidden haemorrhage upon total haemorrhage, MIS-TLIF is comparable with open TLIF. For lumbar degenerative disease, MIS-TLIF owns additional advantage of decreased hidden haemorrhage more than less visible haemorrhage and improved clinical outcomes.
